# Phase I/II study of azacitidine and capecitabine/oxaliplatin (CAPOX) in refractory CIMP-high metastatic colorectal cancer: evaluation of circulating methylated vimentin

**DOI:** 10.18632/oncotarget.11317

**Published:** 2016-08-16

**Authors:** Michael J. Overman, Van Morris, Helen Moinova, Ganiraju Manyam, Joe Ensor, Michael S. Lee, Cathy Eng, Bryan Kee, David Fogelman, Rachna T. Shroff, Thomas LaFramboise, Thibault Mazard, Tian Feng, Stanley Hamilton, Bradley Broom, James Lutterbaugh, Jean-Pierre Issa, Sanford D. Markowitz, Scott Kopetz

**Affiliations:** ^1^ Department of Gastrointestinal Medical Oncology, The University of Texas M. D. Anderson Cancer Center, Houston, TX, USA; ^2^ Department of Medicine and Case Comprehensive Cancer Center, Case Western Reserve University and Case Medical Center, Cleveland, OH, USA; ^3^ Houston Methodist Cancer Center, Houston Methodist Research Institute, Houston, TX, USA; ^4^ Department of Genetics and Genome Sciences, Case Western Reserve University School of Medicine, Cleveland, OH, USA; ^5^ Fels Institute for Cancer and Molecular Biology, Temple University, Philadelphia, PA, USA; ^6^ Department of Bioinformatics and Computational Biology, The University of Texas M. D. Anderson Cancer Center, Houston, TX, USA; ^7^ Division of Pathology and Laboratory Medicine, The University of Texas M. D. Anderson Cancer Center, Houston, TX, USA; ^8^ Division of Hematology/Oncology, Department of Medicine, University of North Carolina, Chapel Hill, NC, USA

**Keywords:** methylation, azacitidine, CIMP, colorectal cancer, vimentin

## Abstract

**Purpose:**

Hypermethylation of promoter CpG islands (CIMP) has been strongly implicated in chemotherapy resistance and is implicated in the pathogenesis of a subset of colorectal cancers (CRCs) termed CIMP-high.

**Experimental Design:**

This phase I/II study in CRC (phase II portion restricted to CIMP-high CRC), treated fluoropyrimidine/oxaliplatin refractory patients with azacitidine (75 mg/m^2^/day subcutaneously D1-5) and CAPOX (capecitibine and oxaliplatin) every three weeks.

**Results:**

Twenty-six patients (pts) were enrolled in this study: 15 pts (12 treated at MTD) in phase I and 11 pts in phase II. No dose limiting toxicities were observed. A total of 14 pts were CIMP-high. No responses were seen. CIMP-high status did not correlate with efficacy endpoints [stable disease (SD) or progression-free survival (PFS)] or baseline vimentin methylation level. Changes in vimentin methylation over time did not correlate with efficacy outcomes. Baseline methylated vimentin correlated with tumor volume (*P*<0.001) and higher levels of baseline methylation correlated with the obtainment of stable disease (*P*=0.04).

**Conclusions:**

Azacitidine and CAPOX were well tolerated with high rates of stable disease in CIMP-high pts, but no objective responses. Serum methylated vimentin may be associated with benefit from a regimen including a hypomethylation agent, although this study is not able to separate a potential prognostic or predictive role for the biomarker.

## INTRODUCTION

Epigenetic modification of gene expression represents a unique mechanism of oncogenic alteration that can lead to widespread changes in gene expression. Suppression of key gene targets such as tumor suppressors, represents an early and critical event in carcinogenesis. [1] DNA methyltransferases are responsible for this process by the addition of a methyl group to the 5′ position of cytosines, generally in the context of “CpG” dinucleotide sequences. Hypermethylation of such sequences in the context of gene promoters results in the suppression of transcription. One established subset of colorectal cancer has been characterized by the presence of aberrant DNA methylation at CpG island promoters (CpG island methylator phenotype, CIMP). In contrast to the majority of CRCs, which demonstrate widespread chromosomal gains and loss, termed chromosomal instability (CIN), the majority of CIMP-high CRCs are characterized by chromosomal stability. [2] In addition the molecular profile of CIMP-high CRC is unique with both a high rate of mismatch repair deficiency, or microsatellite instability (MSI), and RAS pathway activation, KRAS or BRAF mutations. [3, 4] Clinicopathological features are unique for CIMP-high CRC with an association with serrated adenoma precursor lesions, female gender, and proximal colonic location. [4]

In addition to the role of DNA methylation in CRC carcinogenesis, CIMP-high has been correlated with chemotherapy resistance in not only CRC but also other tumor types. [6, 9] The acquired resistance to a variety of chemotherapy agents has been correlated with epigenetic changes in a variety of *in vitro* and *in vivo* models. [10] In colorectal cancer cell lines and xenografts the use of either the hypomethylator agent decitabine or azacitidine was demonstrated to reverse 5-FU resistance. [11, 12] The resistance to platinum-based chemotherapy in ovarian cancer has been correlated with the development of MLH1 methylation, which was demonstrated to be reversible with the use of the demethylating agent decitabine. [13, 14] A recent *in vitro* study has also demonstrated the ability of chemotherapy, and in particular platinum agents, to synergistically increase the hypomethylation of decitabine. [15]

Though single agent activity of hypomethylators in solid tumors as opposed to hematological cancers, has not been well demonstrated, recent clinical trials using epigenetic modulation in combination with chemotherapy have demonstrated the ability to restore chemotherapy sensitivity. [16–18] In particular in one study investigating platinum refractory ovarian cancer, the addition of azacitidine at 75mg/m^2^/days for 5 days in conjuncture with carboplatin every 4 weeks demonstrated partial responses in 4 and stable disease in 10 out of 20 treated patients. [17] More recently the combination of azacitidine and entinostat, a histone deacetylase (HDAC) inhibitor demonstrated a 4% response rate in non-small cell lung cancer, and interestingly 4 of the 19 patients who underwent subsequent anticancer systemic therapy demonstrated objective responses. [19]

We have previously developed a methodology termed methyl-BEAMing that allows for the quantification of methylated DNA fragments from a patients’ blood. [20] When applied to vimentin, a gene preferentially methylated in colorectal cancer, the presence of methylated vimentin (1 fragment in 2 ml of plasma) was strongly correlated with colorectal cancer with an area under the receiver operating curve of 0.95 for patients with stage IV CRC compared to healthy adults. [20–22] Thus, methyl-BEAMing for methylated vimentin represents a quantitative means of interrogating the methylation status of CRC tumor DNA from the peripheral blood.

In this study we hypothesized that the use of azacitidine would restore fluoropyrimidine and oxaliplatin chemotherapy sensitivity, and that this effect would be predominantly seen in CIMP-high CRC, in which DNA hypermethylation is known to be highly active and plays a critical role in carcinogenesis. In addition, we explored the role of methylated vimentin as a potential biomarker for epigenetic modulation in CRC.

## RESULTS

### Baseline characteristics

This study enrolled 26 patients from 8/2010 to 12/2012: 15 in the phase I portion and 11 in the phase II portion, Table 1. No dose-limiting toxicities (DLTs) occurred. Three patients were treated at the starting dose level and 12 at the highest dose level (subcutaneous azacitidine 75mg/m^2^/day days 1 to 5, intravenous oxaliplatin 110mg/m^2^ day 2, and oral capecitabine 1500mg/m^2^ divided twice daily for 2 weeks every three weeks) or maximum tolerated dose (MTD). The additional 6 patients at the MTD were enrolled due to concerns related to the potential for delayed hematological toxicity, which was not observed.

A total of 14 patients (3 patients in the phase I cohort) and 11 patients in the phase II cohort were CIMP-high. CIMP status was unknown in one patient. Prior progression on oxaliplatin-based therapy was retrospectively determined to meet Response Evaluation Criteria in Solid Tumors (RECIST) criteria in 81% of patients. The median number of cycles administered was 4.5 (range: 1-11).

**Table 1:** Baseline characteristics (N=26)

Characteristics	Number of Patients (%)
Median age, years [range]	60, [34-78]
Female Gender	11 (42%)
Poorly differentiated	3 (12%)
ECOG Performance Status 0-1	26 (100%)
CIMP-high	14 (54%)
2 markers	7
3-6 markers	7
Braf V600E mutation	2 (8%)
Kras mutation	17 (65%)
Metastatic sites	
Liver	13 (50%)
Lung	16 (62%)
Peritoneum	6 (23%)
Non-regional lymph nodes	5 (19%)
Median Prior lines of therapy [range]	4.5 [2–9]
Prior oxaliplatin progression	
RECIST progression	21 (81%)
Non-RECIST criteria progression	5 (19%)

### Toxicity

Common grade 3 or 4 toxicities and all toxicities occurring in > 20% of patients are shown in Table 2. The most common grade 3 or 4 toxicities were neutropenia (38%), anemia (12%), diarrhea (12%), and oxaliplatin hypersensitivity reaction (12%).

**Table 2:** Grade 2-4 toxicity

**Hematologic Toxicities**	**Grade 2**	**Grade 3**	**Grade 4**
Neutropenia	4	7	3
Anemia	5	3	
Thrombocytopenia	3	1	
**Non-hematologic Toxicities**	**Grade 2**	**Grade 3**	**Grade 4**
Fatigue	14		
Neuropathy		1	
Nausea	4	2	
Diarrhea	3	3	
Vomiting	2	2	
Oxaliplatin hypersensitivity reaction	1	3	
Anorexia	7	1	
Dehydration	3	2	

### Efficacy

**Figure 1:**
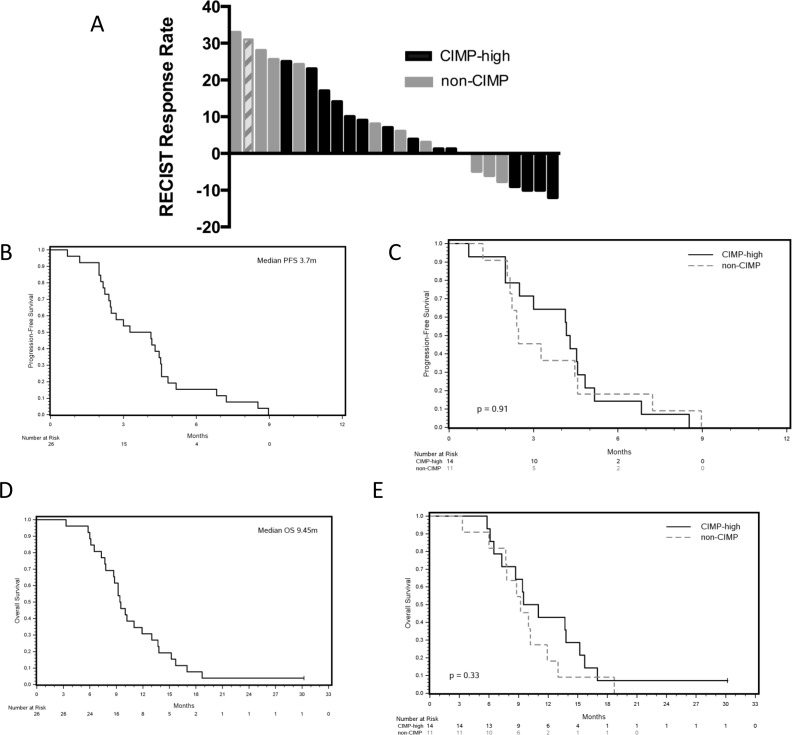
Efficacy results for the combination of CAPOX and azacitidine **A.** Radiographic best response stratified by CIMP status, with single case of unknown CIMP status represented by hashed bars. **B.** Progression-free survival for all patients. **C.** Progression-free survival stratified by CIMP status (*n* = 25). **D.** Overall survival for all patients. **E.** Overall survival stratified by CIMP status (*n* = 25).

All patients were evaluable for response assessment. No responses were seen. The study closed following the enrollment of 11 patients in the phase II cohort due to the prespecified futility boundary required one response in the first 11 treated patients. Stable disease occurred in 17 patients (65%) with a median duration of 4.5 months (range 2.4 to 9m). Stable disease lasting for > 6 months was seen in 4 patients of whom 2 were CIMP-high. Stable disease occurred in 11 CIMP-high and 6 non-CIMP patients, *P* = 0.22. A waterfall plot is shown in Figure 1A and a spider plot in Supplemental Figure 1. The median progression-free survival (PFS) and overall survival (OS) for all patients was 3.7 and 9.5 months, respectively, (Figure 1B + 1D). There was no correlation between CIMP-high status and either PFS or OS, Figure 1C + 1E.

### Post-progression therapy

Post-treatment median OS was 5.7m. Thirteen patients received subsequent post-study treatment with best RECIST response of progressive disease in 11 patients and stable disease in 2 patients (Table 3). The median PFS for these 13 patients was 1.8 months. Of the 4 patients with > 6months of stable disease on study, 2 did not receive subsequent therapy, 1 demonstrated progression to regorafenib, and 1 demonstrated stability to regorafenib lasting 14.6 months.

**Table 3:** Post-progression therapy for 13 patients treated following clinical trial completion

Study Number	CIMP Status	Post-Progression Therapy	Time from Azacitdine study completion to next treatment (days)	Best Response	Time to progression (days)
3	CIMP-High	5-FU + Oxaliplatin + Bevacizumab + Cetuximab	19	PD	61
21	CIMP-High	Erlotinib	1	PD	60
19	CIMP-High	Irinotecan	1	PD	76
24	CIMP-High	Regorafenib	29	PD	46
25	CIMP-High	Regorafenib	22	PD	44
26	CIMP-High	Regorafenib	1	SD	439
11	non-CIMP	AMG 208 (c-MET inhibitor)	32	PD	34
12	non-CIMP	Bevacizumab + Lenalidomide	51	PD	55
7	non-CIMP	Cetuximab + Erlotinib + Bevacizumab	52	SD	111
13	non-CIMP	Erlotinib + Bortezomib	32	PD	60
14	non-CIMP	FOLFOX + Cetuximab + Dasatinib	40	PD	60
6	non-CIMP	GDC-0449 (hedgehog inhibitor)	61	PD	45
9	Unknown	Irinotecan + Bevacizumab + Cetuximab	26	PD	45

### Vimentin methylation correlates with CIMP-high status and azacitdine modulates tumor vimentin methylation *in vitro* and *in vivo*

**Figure 2:**
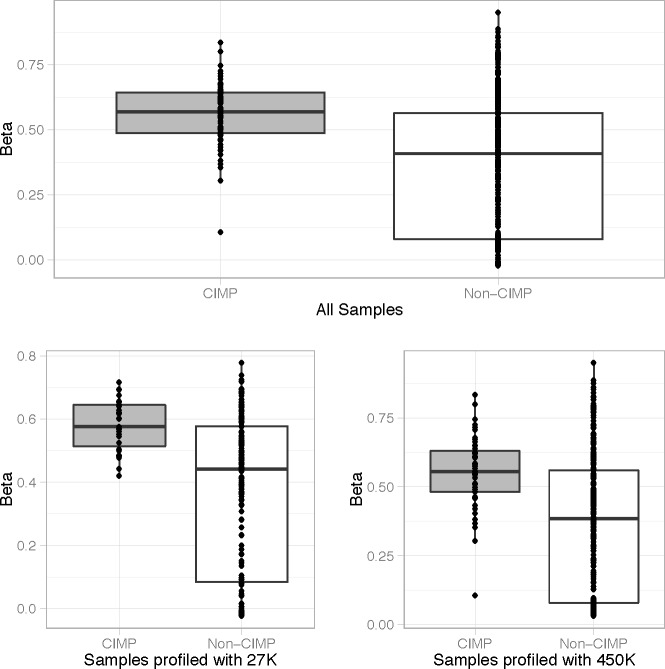
Vimentin methylation correlates with CIMP-high status The percentage of vimentin methylation (Beta-value) for 574 CRC cases (218 with HumanMethylation27 BeadChip and 356 with the HumanMethylation450 BeadChip) from the Cancer Genome Atlas (TCGA) Research Network was statistically correlated with the presence of CIMP-high status (CIMP). All values are shown with the box representing 25-75% percentiles and median represented as a line.

**Figure 3:**
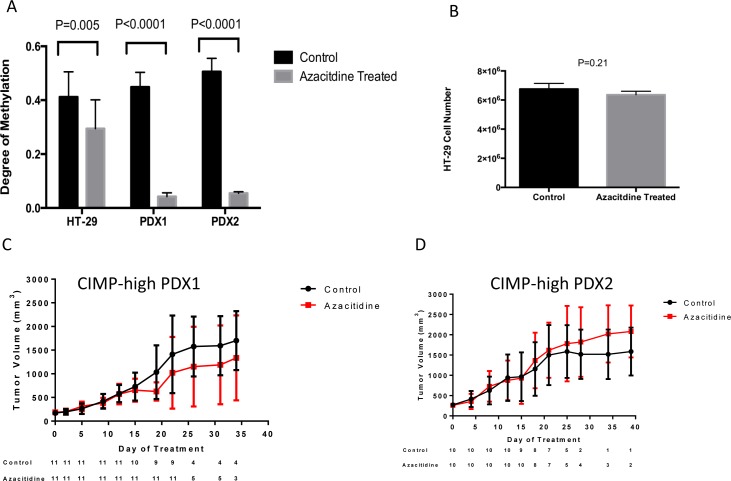
**A.** Azacitidine reduces tumor vimentin methylation but not tumor size in the CIMP-high HT-29 CRC cell line and two CIMP-high CRC patient derived xenografts (PDXs). **A.** Pre and post azacitidine treatment samples were analyzed for the vimentin methylation status by the Illumina Infinium HumanMethylation450 BeadChip array. CIMP-high HT29 CRC cells were treated with and without continuous azacitidine (1 uM) for 2 weeks and then counted. Two CIMP-high PDXs were treated with phospho-buffered saline control or 0.5 mg/kg azacitidine administered intraperitoneal twice weekly for two generations in NOD-SCID-gamma mice, and tumor measurements were recorded twice weekly. **B.** Cell counts for CIMP-high HT-29 CRC cell line with or without azacitidine treatment. **C.-D.** The tumor volume for two CIMP-high PDXs with or without azacitidine treatment. Bars represent 95% confidence intervals.

As the methylation status of vimentin in relation to CIMP-high status has not been evaluated, we evaluated the vimentin methylation from 574 CRC (218 with HumanMethylation27 BeadChip and 356 with the HumanMethylation450 BeadChip) samples from the Cancer Genome Atlas (TCGA) Research Network. Although vimentin methylation was detected in the majority of colon cancers we found a statistically significant increase in level of methylated vimentin in patients with CIMP-high CRC, *P* < 0.001 (Figure 2). For all samples the median Beta value for vimentin methylation was 0.58 for CIMP-high patients and 0.41 for non-CIMP patients. To determine the ability of azacitidine to modulate vimentin methylation levels we treated the CIMP-high CRC cell line, HT-29, and two CIMP-high CRC patient derived xenograft (PDX) samples. In all cases a significant reduction in tumor methylated vimentin was seen between treated and non-treated samples, *P* < 0.01 (Figure 3A). However, despite vimentin methylation modulation, HT-26 cell count and PDX tumor volume did not show statistical reductions with single agent azacitidine treatment (Figure 3B-3D).

### Baseline methylated vimentin correlates with tumor volume and tumor response

**Figure 4:**
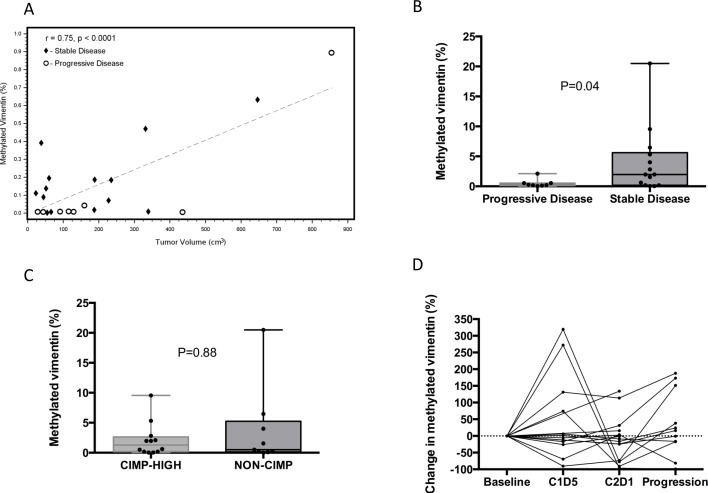
Correlation of baseline circulating methylated vimentin with tumor volume and tumor response status **A.** Correlation plot between tumor volume and percent circulating methylated vimentin. Tumor volume statistically correlated with percent circulating methylated vimentin. The dashed line represents the line of best fit from ordinary least squares regression model with a Pearson correlation coefficient of 0.75. Diamonds represent stable disease patients and circles represent progressive disease patients. **B.-C.** The baseline percent circulating methylated vimentin normalized by baseline tumor volume was stratified by CIMP status and tumor response to CAPOX and azacitidine, respectively. All values are shown with the box representing 25-75% percentiles and median represented as a line. Two-tailed paired t test use for p-value (*N* = 22). **D.** Individual patient data for the percent of circulating methylated vimentin for cases with baseline methylation > 1% (*N* = 15).

A baseline plasma sample was available for 25 patients and the mean, median, and range of methylated vimentin were 4.2%, 14.2%, and 0.1% to 89.3%, respectively. A total of 10 patients had baseline levels < 1% (3 with CIMP-high and 6 with non-CIMP, 1 with CIMP status unknown). Total baseline tumor volume was calculated for 23 patients; volumes could not be calculated for 3 patients with peritoneal carcinomatosis. Baseline plasma methylated vimentin correlated with tumor volume, R = 0.75, *P* < 0.0001, Figure 4A. After normalizing baseline vimentin methylation by tumor volume, increased baseline plasma methylated vimentin showed a statistically significant correlation with the occurrence of disease stability on therapy, *P* = 0.04, Figure 4B

Baseline plasma methylated vimentin did not correlate with overall survival, *P* = 0.74. Adjusting for tumor volume there was no correlation between baseline plasma methylated vimentin and tumor CIMP status, *P* = 0.88. Figure 4C.

### Changes in methylated vimentin over time

The percent change in methylated vimentin during therapy for the 15 patients with baseline values > 1% is shown in Figure 4D. Patients with baseline methylated vimentin < 1% were more likely to have progressive disease as best response, although the trend was not significant, *P* = 0.09. Methylated vimentin decreased in 9 of 15 patients at C2D1 with a median reduction of 9.2% (range: -96.3% to 134.2%). Changes in methylated vimentin from baseline and either C1D5 or C2D1 did not correlate with disease stability. A reduction in methylated vimentin at C2D1 did correlate with a reduction in global DNA methylation at both C1D5 (−8.8% *vs*. -1.5%, *P* = 0.01) and C2D1 (−2.5% *vs*. -0.2%, *P* = 0.05). At the time of progression methylated vimentin was increased in 9 of 15 patients (median 15.3%, range: -99.3% to 2068%). Interestingly all 6 patients with an initial increase in methylated vimentin at C1D5 demonstrated stability, whereas 3 of 6 patients with a decrease at C1D5 demonstrated best response of progression.

### CAPOX/Azacitdine demethylates peripheral blood mononuclear cells (PBMCs)

Global DNA methylation was reduced from pre to post-treatment at all timepoints. The greatest reductions occurred at the day 5 timepoint for cycle 1 (median -8%, range -18.3% to 3.3%) and cycle 2 (median -7.4%, range -21.2% to 4%). The reduction at C2D1 was less with a median reduction of 0.6% (range -6.6% to 5.6%). Changes in global methylation did not correlate with the chance of obtaining SD at any time point.

## DISCUSSION

This study reports for the first time the results of combining azacitidine with a standard cytotoxic chemotherapy combination for colorectal cancer, CAPOX. The results show the safety of this combination and note a high rate of stable disease. More interestingly this study investigated a possible blood based epigenetic biomarker, methylated vimentin, and suggests that baseline circulating levels of tumor methylation may represent a prognostic and/or predictive biomarker.

Biomarkers for epigenetic therapy in solid tumors have remained elusive with a number of studies finding minimal to no correlation between peripheral mononuclear cell methylation status and tumor methylation status. [23, 24] In one particular study investigating the effects of decitabine in solid tumors, pre and post global DNA methylation of CpG islands demonstrated no correlation between PBMC and tumor samples, r = 0.22, and no correlation for the change in DNA methylation, r = 0.02. [23]

In this study we chose to use a gene, vimentin, in which increases in methylation have demonstrated a strong correlation with the development of colon cancer, and that has been used as the target for a commercialized fecal DNA methylation diagnostic test (ColoSure, LabCorp). Our findings demonstrate that baseline increased circulating methylated vimentin correlates with greater tumor bulk. In addition, circulating methylated vimentin adjusted for radiographic tumor bulk correlated with a higher likelihood of obtaining stable disease. Though limited by sample size, this finding suggests that CRC with greater methylation extent, using vimentin methylation as the surrogate, appear to be more responsive to the combination of chemotherapy and a hypomethylating agent. Despite our hypothesis that this biomarker would demonstrate higher levels of methylation in patients with CIMP-high status, this was not seen in our clinical trial patient samples. Such findings suggest that methylated vimentin is not exclusively a CIMP-high phenomenon. These findings agree with the use of methylated vimentin as a marker across all CRC subtypes.

This study did not find evidence to support the use of plasma methylated vimentin as a pharmacodynamic marker for azacitidine. In part, this reflects the small sample size within this study, only 15 patients had baseline samples with methylated vimentin at > 1%, and the large variations in methylated vimentin that were seen over time. Prior studies investigating pre and post tumor biopsies changes in promoter gene methylation have not correlated with efficacy outcomes. [23–26] These findings and our finding may reflect the challenge of drug delivery in solid tumors and the S-phase specific action of hypomethylating agents, in which incorporation into DNA is limited to only those tumors with high proliferation. In addition, our findings are confounded by the role of increased methylated vimentin as both a marker of tumor burden and likely also a marker of tumor cytotoxicity. A limited number of clinical trials have investigated either global or gene specific DNA methylation changes from plasma or serum as potential pharmacodynamic biomarkers of epigenetic therapy. [16, 19, 25] Though changes in methylation were seen in all studies, occurring in 100%, 50%, or 38% of cases, respectively, only one study found a correlation between gene hypomethylation and clinical outcomes. In this clinical trial investigating azacitidine and entinostat in non-small cell lung cancer 10 out of 26 patients demonstrated promoter hypomethylation of at least 2 out of 4 target genes. [19] In these 10 patients both PFS and OS were statistically improved.

Two recent clinical trials have suggested improved chemotherapy and targeted therapy responsiveness following epigenetic therapy. [19, 27] In contrast to these two reports we did not see any evidence for increase activity following our study with 11 of 13 patients demonstrating progression on their next line of therapy.

In this study azacitdine was dosed as 75mg/m^2^ daily for 5 days every three weeks, which is similar to the current approved dose in myelodysplastic syndrome of 75mg/m^2^ daily for 7 days every 4 weeks. The optimal dose and dosing schedule for azacitdine in solid tumors has not been identified, but both *in vitro* and clinical data have demonstrated that lower drug levels are able to provide adequate hypomethylation and clinical benefit in hematological malignancies. [28–30] Whether improved hypomethylation and greater clinical activity would have been seen from a lower more continuous dosing schedule is not known. In addition the lack of on-treatment tumor biopsies represents a limitation of this study. Despite the preclinical data demonstrating clear modulation of vimentin with azacitdine, it is still unclear the impact of azacitidine on actual tumor tissue.

In conclusion we demonstrate the safety of combining azacitidine with capecitabine and oxaliplatin in colorectal cancer. This clinical trial did not validate the use of CIMP-high status as a predictive biomarker for azacitdine. In addition evidence to support the use of plasma methylated vimentin as a pharmacodynamic marker for azacitdine was not seen. Pre-treatment circulating methylated vimentin correlated with both tumor bulk and the obtainment of clinical benefit for study treatment. Further work exploring both alternative epigenetic schedules and combinations, and the use of methylated vimentin as a predictor for epigenetic benefit in CRC are needed.

## MATERIALS AND METHODS

### Patients

This is an open-label, single-institution, phase I/II study of azacitidine and capecitabine and oxaliplatin (CAPOX) for histologically confirmed colorectal adenocarcinoma. Eligibility criteria required Eastern Cooperative Oncology Group (ECOG) performance status of ≤ 2, adequate organ function, measurable disease, peripheral neuropathy ≤ 2 grade 2, and refractory to fluoropyrimidine and oxaliplatin therapy (defined as either clinical or radiographic progression on or within 3 months of treatment). In addition for the phase II portion, enrollment was restricted to CIMP-high patients, which was defined as hypermethylation at ≥ 2 of 6 methylation-specific polymerase chain reaction (PCR) markers (hMLH1, P16, P14, MINT1, MINT2, and MINT31). CIMP testing was performed at University of Texas MD Anderson Cancer Center (UTMDACC) in a CLIA-certified manner (Supplemental Table 1). [7] This study was conducted with approval by the UTMDACC Institutional Review Board, and all participants provided written informed consent (NCT01193517).

### Study design and treatment

A 3+3 design was utilized for the phase 1 portion followed by a phase II expansion at the MTD. The starting dose level was subcutaneous azacitidine 75mg/m^2^/day days 1 to 5, intravenous oxaliplatin 90mg/m^2^ day 2, and oral capecitabine 1500mg/m^2^ divided twice daily for 2 weeks every three weeks. A single dose escalation level (+1) existed, in which oxaliplatin was increased to 110mg/m^2^. Restaging was conducted every three cycles.

Dose limiting toxicities (DLTs), according to the National Cancer Institute Common Terminology Criteria for Adverse Events Version 4.0, were defined as any of the following toxicities that occurred during the first treatment cycle: drug related ≥ grade 3 non-hematological toxicity (excluding correctable grade 3 nausea, vomiting, diarrhea, electrolyte, and glucose abnormalities), grade 4 anemia, grade 4 thrombocytopenia, grade 4 neutropenia for > 5 days, ≥ grade 3 febrile neutropenia, or a treatment delay of > 14 days.

### Cell line and patient derived xenograft (PDX)

The CIMP-high colorectal cancer cell line HT29 (from the American Type Culture Collection) was maintained in DMEM-F12 supplemented with 10% fetal bovine serum and 2 mM L-glutamine. [31] Short tandem repeat DNA profiling was done approximately 6 months prior to treatment to confirm HT-29 identity. The cells were seeded in 6 well plates, allowed to attach for 24 h at 37°C. Thereafter half HT29 cells were continuously exposed to 1 uM azacitidine for 2 weeks. During treatment, azacitidine (Sigma-Aldrich) was freshly prepared daily with new growth medium at 1 uM and the growth medium was replaced with new medium with (treated cells) or without (parental cells) azacitidine. The cells were subcultured until the attached cells reached 70% confluence. At the end of treatment, cells were counted and collected for DNA. All analyses were done in triplicate.

Biopsy specimens from tumors of two separate patients with MLH-1 methylated CIMP-high metastatic colorectal cancer were implanted into the subcutaneous flanks of female NOD-SCID-gamma mice in order to establish PDX models. Upon successful uptake of the tumor, mice were randomized in an initial generation to receive a phospho-buffered saline control or 0.5 mg/kg azacitidine administered intraperitoneal twice weekly. Once the tumor volume reached 1500 mm3 in size, mice were sacrificed, and the tumors were split and expanded into a second generation of female NOD-SCID-gamma mice in order to maximize the exposure time of the demethylating agent to the tumor. Mice continued to receive phosphate-buffered saline control or azacitdine (0.5 mg/kg) according to the treatment administered for the prior generation of the given tumor. Tumor measurements were recorded twice weekly. At the end of treatment each arm had at least 3 separate tumors for final tumor size assessment and DNA collection.

### Tumor methylation analysis

The CIMP and vimentin methylation status for 574 CRC cases (218 with the Illumina Infinium HumanMethylation27 BeadChip and 356 with the Illumina Infinium HumanMethylation450 BeadChip) from TCGA Research Network (http://cancergenome.nih.gov/) were analyzed. The sequences within the human genome that corresponded to the PCR primers for the 6 CpG islands analyzed in the clinical trial (hMLH1, p16, p14, MINT1, MINT2, and MINT31) were identified using BiSearch (http://bisearch.enzim.hu/). [32, 33] Using NCI Blast the corresponding nucleotide locations within the Genome Reference Consortium Human genome build 37 (GRCh37) were correlated with the CpG islands from the HumanMethylation450 array (Supplemental Table 2A-B). Within the PCR amplified regions of hMLH1, MINT2, and MINT31, 1-2 CpG islands were identified, while for MINT1, p14, and p16 1-2 CpG islands were only identified < 300 nucleotides adjacent from each of the primer pairs. For each CpG island the beta-value was converted to an M-value and the distribution of M-values from the TCGA dataset was plotted and found to be bimodal (Supplemental Table 3A-B). [34] The rate of methylation positivity for each marker was found to correlate well with clinically obtained CIMP testing and > 2 methylated markers was used to define CIMP-high. CpG islands from the HumanMethylation27 BeadChip could not be aligned with the clinical CIMP PCR primers, and thus for this cohort a clustering method of methylation profiles was used as previously described. [35]

Pre and post azacitidine treatment samples from HT-29 and the two PDX samples were analyzed by the Illumina Infinium HumanMethylation450 BeadChip array and methylated vimentin status determined by analysis of the three methylation probes correlating with exon 1 of vimentin. Methylation of exon 1 was chosen to align with prior work investigating vimentin methylation status. [20–22]

### Plasma vimentin and peripheral blood mononuclear cell (PBMC) methylation analyses

Blood samples were collected at baseline, cycle 1 day 5 (C1D5), cycle 2 day 1 (C2D1), cycle 2 day 5 (C2D5), and at each restaging. Samples were processed within 2 hours in Cell Preparation Tubes (BD, Franklin Lakes, NJ). Plasma and pbmcs were separated after a single centrifugation step and stored at -80°C until analysis.

For circulating cell-free vimentin methylation DNA was purified and extracted from 2 ml plasma aliquots. Methylated vimentin status of exon 1 was determined by bisulfite conversion, PCR amplification of the vimentin differentially methylated domain with bisulfite specific methylation indifferent primers [20], and analysis of the methylation status of the amplicon by Next Generation bisulfite sequencing. Individual sequence reads were classified as methylated if methylation was detected at greater than or equal to 5 of the 10 CpGs on the amplicon, and the percent of methylated vimentin reads in circulating DNA was determined for each sample.

For global DNA methylation of PBMC, DNA was isolated and treated with bisulfite. LINE1 methylation assay coupled with pyrosequencing was then utilized. [36, 37].

### Statistical analysis

The phase I primary endpoint was to determine MTD and the phase II primary endpoint was response rate by RECIST v1.1. The phase II sample size was 30 patients to ensure that the posterior 90% credibility interval will be (11%, 35%), under the assumption of a 20% response rate. Phase II early stopping rules required ≥ 1 response in the first 11 patients.

In a retrospective manner the previously determined oxaliplatin progression prior to study entry and post-study treatment response were determined by RECIST v1.1 criteria. Tumor volumes were calculated using computed tomography scans using Voxtool (version 3.0.64z, General Electric Corp., Fairfield, CT).

Descriptive statistics for continuous (mean, median, and range) and categorical variables (frequency and percentage) are reported. Fisher's exact test and Wilcoxon rank-sum test were used for categorical and continuous variables, respectively. Ordinary least squares regression and Pearson's product-moment correlation coefficient were used to assess linear relationships. Survival was estimated using the Kaplan-Meier method. The two-sided log-rank test was used to assess group differences in survival endpoints. All P values presented are two-sided and statistical significance is *P* ≤ 0.05.

## SUPPLEMENTARY MATERIAL TABLES AND FIGURE


