# Supplementary material for: Phase I/II study of azacitidine and capecitabine/oxaliplatin (CAPOX) in refractory CIMP-high metastatic colorectal cancer: evaluation of circulating methylated vimentin
Source: Oncotarget. 2016 Aug 16;7(41):67495–506. doi: 10.18632/oncotarget.11317 (PMC5341892; doi:10.18632/oncotarget.11317)
Supplement: Supplementary file 1 [file oncotarget-07-67495-s001.pdf]

Phase I/II study of azacitidine and capecitabine/oxaliplatin (CAPOX) in refractory CIMP-high metastatic colorectal cancer: evaluation of circulating methylated vimentin

Supplementary Material

Supplementary Table 1: CLIA certified PCR primers used for bisulfite sequencing to determine CIMP status

CIMP Locus	Forward Primer	Reverse Primer	Sequencing Primer
MLH1	5′-TTG GTA TTT AAG TTG TTT AAT TAA TAG TTG-3′	5’-AAA ATA CCT TCA ACC AAT CAC CTC-3’	5’-AGT TAT AGT TGA AGG AAG AA-3’
MINT1	5’-GGT TTT TTG TTA GYG TTT GTA TTT-3’	5’-ATT AAT CCC TCT CCC CTC TAA ACT T-3’	5’-TTT AGT AAA AAT TTT TTG GG-3’
MINT2	5’-AGT GTT AGA AAA ATG TGT TG-3’	5’- CTA CAA TTA AAC ATC AAT TAT AT-3’	5’-GAA TTT TAG TAT TTA AGT T-3’
MINT31	5’-TGT TTT TTA YGY GTG TAT A-3’	5’-AAT CCC ACA ACT TTC TAA AAT A-3’	5’-TTT AGT TTT TTT TTG-3’
p14	5’-TTA GTT TGT AGT TAA GGG GGT AGG AG-3’	5’-AAA AAT CAC CAA AAA CCT AC-3’	5’-TTT ATT TTT GGT GTT AAA GG-3’
p16	5’-GGT TGT TTT YGG TTG GTG TTT T-3’	5’-ACC CTA TCC CTC AAA TCC TCT AAA A-3’	5’-TTT TTG TTT GGA AAG AT-3’

Supplementary Table 2A: Genomic locations in GRCh37.p13 corresponding to the PCR primers used for CLIA certified CIMP testing

CIMP Locus	Chromosome	Start (GRCh37.p13)	End (GRCh37.p13)	Sequencing Start
MLH1	3	37034879	37034990	37034936 (>End)
MINT1	5	75379990	75380112	75380029 (>End)
MINT2	2	58655009	58655149	58655104 (>Start)
MINT31	17	48636910	48636784	48636870 (>Start)
p14	9	21994309	21994426	21994394 (>Start)
p16	9	21974960	21975105	21975011 (>Start)

Supplementary Table 2B: CpG islands found in the Illumina Infinium HumanMethylation450 either lying within the bisulfite sequencing primers or in closest distance, determined by number of nucleotides separating the locus and the start or end of sequenced locus.

Clinical CIMP	CpG island	Chromosome	GRCh37.13 site	Distance
MINT2	cg26154670	2	58655041	within sequenced site
MINT2	cg09859179	2	58655104	within sequenced site
MLH1	cg03192963	3	37034909	within sequenced site
MLH1	cg06791151	3	37034956	within sequenced site
MINT31	cg24280645	17	48636900	within sequenced

				site
MINT1	cg10583931	5	75379800	190
p16	cg13601799	9	21974704	256
p14	cg07562918	9	21994435	41
p14	cg03079681	9	21994223	86

Supplementary Table 3A: Threshold values used to dichotomize methylation at CpG islands based on Illumina Infinium HumanMethylation450 BeadChip results

Gene	CpG island	M-value	Beta-value
p14	cg03079681	-1.752	0.229
p14	cg07562918	-1.321	0.286
p16	cg13601799	-1.788	0.225
MINT1	cg10583931	-2.279	0.171
MINT2	cg09859179	-2.214	0.177
MINT2	cg26154670	-2.587	0.143
MINT31	cg24280645	-3.296	0.092
MLH1	cg03192963	-2.826	0.124
MLH1	cg06791151	-3.213	0.097

Supplementary Table 3B: Rates of methylation positivity of each gene marker in the TCGA HumanMethylation450 BeadChip samples and comparison to CLIA certified CIMP testing, as utilized for clinical trial enrollment, in 440 stage IV CRC patients who were tested for CIMP status under a screening protocol from 8/2010 to 10/2013.

		% Positive (CIMP from 440 patient clinical cohort)	% Positive (TCGA)
p14	Either cg03079681 or cg07562918	26.1	32.9
p16	cg13601799	22.2	32.6
MINT1	cg10583931	19.9	34.0
MINT2	Both cg09859179 and cg26154670	18.4	21.3
MINT31	cg24280645	31.0	35.4
MLH1	Either cg03192963 or cg06791151	8.0	11.5
CIMP-High	≥3/6 markers	19.9	27.2

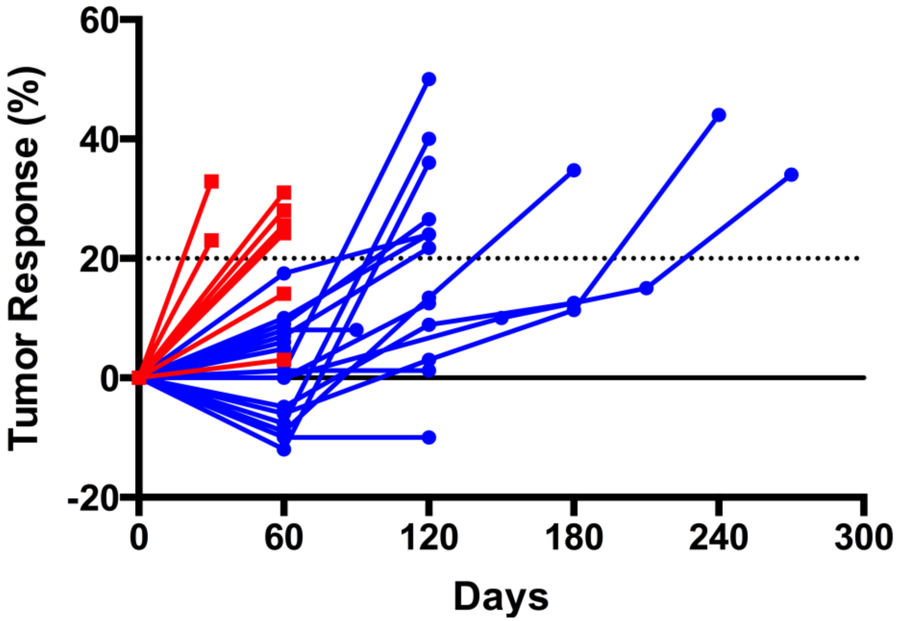

Supplementary Figure 1: Spider plot of all patients treated with CAPOX and azacitidine. Red color represents best response of progression and blue color represents best response of stable disease.
